# Computational Offloading in Mobile Edge with Comprehensive and Energy Efficient Cost Function: A Deep Learning Approach

**DOI:** 10.3390/s21103523

**Published:** 2021-05-19

**Authors:** Ziaul Haq Abbas, Zaiwar Ali, Ghulam Abbas, Lei Jiao, Muhammad Bilal, Doug-Young Suh, Md. Jalil Piran

**Affiliations:** 1 Faculty of Electrical Engineering, GIK Institute of Engineering Sciences and Technology, Topi 23640, Pakistan; ziaul.h.abbas@giki.edu.pk; 2 Telecommunications and Networking Research Center, GIK Institute of Engineering Sciences and Technology, Topi 23640, Pakistan; zaiwar.ali@giki.edu.pk; 3 Faculty of Computer Science and Engineering, GIK Institute of Engineering Sciences and Technology, Topi 23640, Pakistan; abbasg@giki.edu.pk; 4 Department of Information and Communication Technology, University of Agder (UiA), 4898 Grimstad, Norway; lei.jiao@uia.no; 5 Department of Computer Engineering, Hankuk University of Foreign Studies, Yongin-si 17035, Korea; 6 Department of Electronics and Software Convergence, Kyung Hee University, Yongin-si 17035, Korea; 7 Department of Computer Science and Engineering, Sejong University, Seoul 05006, Korea; piran@sejong.ac.kr

**Keywords:** mobile edge computing, computational offloading, deep learning, cost function, remote execution, energy efficiency

## Abstract

In mobile edge computing (MEC), partial computational offloading can be intelligently investigated to reduce the energy consumption and service delay of user equipment (UE) by dividing a single task into different components. Some of the components execute locally on the UE while the remaining are offloaded to a mobile edge server (MES). In this paper, we investigate the partial offloading technique in MEC using a supervised deep learning approach. The proposed technique, comprehensive and energy efficient deep learning-based offloading technique (CEDOT), intelligently selects the partial offloading policy and also the size of each component of a task to reduce the service delay and energy consumption of UEs. We use deep learning to find, simultaneously, the best partitioning of a single task with the best offloading policy. The deep neural network (DNN) is trained through a comprehensive dataset, generated from our mathematical model, which reduces the time delay and energy consumption of the overall process. Due to the complexity and computation of the mathematical model in the algorithm being high, due to trained DNN the complexity and computation are minimized in the proposed work. We propose a comprehensive cost function, which depends on various delays, energy consumption, radio resources, and computation resources. Furthermore, the cost function also depends on energy consumption and delay due to the task-division-process in partial offloading. None of the literature work considers the partitioning along with the computational offloading policy, and hence, the time and energy consumption due to task-division-process are ignored in the cost function. The proposed work considers all the important parameters in the cost function and generates a comprehensive training dataset with high computation and complexity. Once we get the training dataset, then the complexity is minimized through trained DNN which gives faster decision making with low energy consumptions. Simulation results demonstrate the superior performance of the proposed technique with high accuracy of the DNN in deciding offloading policy and partitioning of a task with minimum delay and energy consumption for UE. More than 70% accuracy of the trained DNN is achieved through a comprehensive training dataset. The simulation results also show the constant accuracy of the DNN when the UEs are moving which means the decision making of the offloading policy and partitioning are not affected by the mobility of UEs.

## 1. Introduction

Computational capabilities of user equipments (UEs) have increased over recent years. However, UEs still have limited computational and battery resources due to the complex and energy-hungry applications [1,2,3]. The spectacular growth of the mobile devices, the massive demands of resource-hungry, and delay-sensitive critical applications, such as voice recognition, real time speech recognition, virtual reality, interactive gaming, augmented reality, video transformation, and content-based image recovery applications have attracted significant attention from researchers working on 5G and beyond networks. The delay sensitive nature of these applications has resulted in an increasingly high computing demand and energy consumption. Therefore, to reduce the energy consumption and service delay of UEs, a new paradigm, known as mobile edge computing (MEC) has been introduced [4,5]. MEC offers computing power and storage capacity to UEs at the edge of wireless networks. In MEC, the UEs offload the compute-intensive and delay-sensitive applications to the mobile edge server (MES) through wireless communication to minimize the serving delay and energy consumption of UEs as it is difficult for an UE with limited computation and storage resources to meet the requirements of such compute-intensive applications. Similarly, battery lifetime is the main constraint of UEs and with local computing UEs may not have better quality of experience.

There are two main categories of computational offloading, namely, total offloading and partial offloading [6,7]. In a total offloading technique, the whole task is offloaded to MES for execution while in partial offloading the task is first divided into different components, and then some of the components are executed locally on UE and some are offloaded to MES for execution. For example, if a task is divided into *n* components, there are $2^{n}$ possible options for *n* components to be executed locally on UE or remotly on MES. Extensive research has been done on partial offloading to answer the question as to how the task components can be divided efficiently, between UEs and MES, to reduce the energy consumption and delays of UEs.

In partial offloading, the task-division-process is important and needs to be considered in the cost function for finding the best option for offloading [8]. Based on the type of applications, partitioning offloading can be classified as data-oriented partitioning offloading (DOPO), continuous-execution partitioning offloading (CEPO), and code-oriented partitioning offloading (COPO) [9]. In this paper, we consider DOPO, which means that an application can be split into components of any size. The size of each component can be selected in order to reduce the service delay and energy consumption of UEs because the number of components per task and the size of each component directly depend on the service delay and energy consumption of UEs [9]. Most of the related work ignores the task partitioning and considers the size of each component as a random variable. However, there are ${}^{m}\;Z_{n}$ number of possible options to divide a single task of size *m* into *n* number of components. For example, a 400-MB task can be divided into three components of: [100,100,200] MB or [200,200,0] MB or [100,300,0] MB or [400,0,0] MB. Therefore, there are four possible ways, i.e., ${}^{400}\;Z_{3}=4$ for 400 MB task to be divided into three components. The number of possible ways of task partitioning also depends on the minimum allowable size of a component, known as division resolution. The value of ${}^{m}\;Z_{n}$ increases exponentially with the task size.

To find the best partitioning in ${}^{m}\;Z_{n}$ possible options and best offloading in $2^{n}$ possible options, the complexity becomes ${}^{m}\;Z_{n}2^{n}$. For m=1 GB and n=10, there are 530×1024=542,720 possible options to divide in and offload components. To avoid this huge computation overhead, in this paper, we introduce a comprehensive cost function for partial offloading and consider all possible partitioning of a computational task to generate a training dataset with minimum cost. The training dataset is used for a supervised deep learning approach to find the best partitioning and offloading policy simultaneously.

The proposed work considers the partitioning process in a partial offloading technique and calculates cost for each possible partitioning and offloading policy and then select the partitioning and offloading policy with minimum cost. Therefore, the energy consumption and execution delay are minimum with a high algorithm complexity. To avoid a high algorithm complexity, we use a supervised deep learning approach to make the decision making process faster and energy efficient. The simulation results show that the proposed work consumes less energy with faster execution in MEC networks.

### 1.1. Related Work

A detailed survey is presented on the evolving multimedia internet-of-things (M-IoT) in [10]. The authors promote several innovative applications, aiming to improve the quality of life by connecting numerous smart devices through emerging enabling technologies. The main focus of the authors is to highlight the overview of M-IoT and the importance of M-IoT applications. Major problems while designing M-IoT networks architecture, protocols, and computing schemes are explored to provide stable IoT architecture. Similarly, a comprehensive survey is presented on the secure deployment of MESs for MEC networks in [11]. The concepts and applications of total offloading and partial offloading techniques are presented in [12,13,14,15], respectively.

The Markov decision process (MDP) is used to solve the offloading time optimization problem in [16]. The authors present computational offloading in MEC as an optimization problem and investigate the optimal offloading MES selection strategy. The authors also consider in the MDP framework, user mobility and heterogeneity of MESs jointly. The value iteration algorithm is used to solve the MDP and obtain the optimal offloading time. The problems of reliability-aware optimal computing offloading and resources allocation are considered in [17]. The authors consider a multi-user, multi-server scenario with limited resources of UEs. They present the computational offloading and allocation problem as a combinatorial optimization problem while considering offloading valuable basic (OVB) constraints. The paper also proposes a task merging strategy to reduce the complexity of the algorithm. The authors in [18,19] consider the traditional optimization technique and game theoretic approach, respectively, for partial offloading in MEC to find the optimal offloading policy. However, the main issue of these techniques is the high complexity of algorithms, which makes their deployment impractical in MEC environment. The wireless energy transper concept is investigated in [20,21,22] to minimize the energy consumption of UEs through proper energy harvesting techniques. In their work, in a single time slot the protocol of first-harvest-then-offload is utilized. Based on the computation rate, a maximization problem is studied in [23] for the decrease in propagation loss that severely affects the harvested energy and computation performance of UE. The enhancement in the computational performance of active UEs by the user cooperation technique is investigated in [24], where the inactive UEs use their harvested energy for the help of active ones. For the computational offloading policy, the authors in [24] consider frequency division multiple access (FDMA) to improve the computation rate. A summery of different techniques in related work is given in Table 1.

The maximum-minimum energy efficiency optimization problem (MMEP) with the joint optimization of energy consumption, time slots for computational offloading and energy transfer, and transmit power at a HAP in WP-MEC system are focused on in [25], and by the application of block coordinate descent (BCD) and fractional programming theory, the authors present algorithms with lower complexity. In [26], the computational energy efficiency of an entire system is improved by the joint consideration of the optimal allocation for UE’s transmit power, CPU frequency, and time for transmission in a WP-MEC system. In [27], the maximization problem of system energy efficiency by the joint consideration of optimal time allocation, local computing capacity, energy consumption, and application offloading is discussed. In [28], the authors consider a stochastic method for battery management and resource allocation decisions in a time slot and propose an algorithm derived from the Lyapunov optimization technique. In [29], the authors utilize Lyapunov optimization for the evaluation of tradeoff between the delay and energy efficiency of a multi-user WP-MEC system. In all the above mentioned work, the improvement in the computation rate is mainly focused. However, the battery life of UE is ignored and the number of sub-tasks in which an application should be divided for partial offloading is assumed to be a fixed value. The authors in [30] investigate the computing problem in ultra-dense cellular networks with a multi-user, multi-server scenario. The problem is divided into two phases, i.e., selection of MES and offloading decision. In the first phase, the UEs are grouped with one MES on the basis of physical distance and workload. While in the second phase, a distributed offloading strategy based on the genetic algorithm is proposed to get an adaptive offloading decision. The authors in [31] investigate the computational offloading of DNN-driven AI applications in 5G-enabled MEC networks. The authors consider dynamic task offloading and propose an online algorithm to reduce the energy consumption of UEs and MESs. The authors in [32,33] present a theoretical model for partial offloading in MEC, only for divisible application. They assume that the components of the task can be executed in parallel. To minimize UE energy consumption, a game-theoretic approach is presented in [34].

A deep learning-based MDP technique is presented in [35]. The authors propose a gradient-based deterministic policy for computational offloading in MEC networks to solve the optimization problem. This work considers a dense distributed cellular network with multi-user, multi-server, and multi-tasks scenario. The authors consider the mobility of MESs and UEs to propose an area-based parallel task offloading model to achieve low latency for delay sensitive applications. Similarly, the authors in [36] also propose a deep reinforcement learning approach in a dynamic MEC networks. The paper optimizes the MES selection for offloading and computing power allocation jointly. Deep learning approaches are considered in [37,38,39,40] to minimize the service delay of the UE. Furthermore, the authors in [41] propose a deep imitation learning technique to minimize the service delay of MDs. The main focus is on the service delay, therefore, their cost function only depends on execution and transmission delays. However, for a better quality of service, it is necessary to take an accurate decision about the offloading policy and component size. Most of the related work considered either service delay or energy consumption or both as a cost function. To the best of our knowledge, in the cost function, there has been no consideration of task-division-process of getting different components of a task.

### 1.2. Novelty and Contributions

The main contributions of the paper are summarized as follows.

- We propose the partitioning process, for the first time, in fine-grained computational offloading in MEC. The proposed work considers the cost of partitioning a task into multiple components and selects the possible partitioning option with minimum cost in all possible partitioning options;
- We combine the selection of task partitioning from ${}^{m}\;Z_{n}$ possible options and partial offloading policy from $2^{n}$ possible options and model as a multi-label classification problem. The computational overhead of finding minimum cost in terms of energy consumption and execution delay while considering the offloading policy and partitioning simultaneously becomes $O({}^{m}\;Z_{n}2^{n})$. Therefore, to avoid this huge computation complexity, we propose a supervised deep learning approach to solve both problems simultaneously with a complexity of trained DNN of O(1). We formulate a comprehensive cost function, which considers multiple parameters, namely, network fluctuations and computing resources of MESs, propagation delay, the time delays, and energy consumptions due to partitioning, transmission, execution, and reception;
- Through extensive simulation results we demonstrate the superiority of the proposed technique, compared with total offloading technique (TOT), random offloading technique (ROT), deep learning-based offloading technique (DOT), and energy efficient deep learning-based offloading technique (EEDOT), in terms of energy consumption and execution delay of UEs;
- The UEs can use the trained DNN to find the offloading policy and partitioning for *n* number of components with minimum cost. Since the cost function depends on both energy consumption and time delay, therefore, the end-user will consume minimum energy with faster decisions on selecting the best partitioning and offloading policy for *n* number of components per task.

The rest of the paper is organized as follows. In Section 2, we present the system model. Section 3 presents the proposed technique. Simulation results are presented in Section 4, and Section 5 concludes the paper.

## 2. System Model

**Figure 1:**
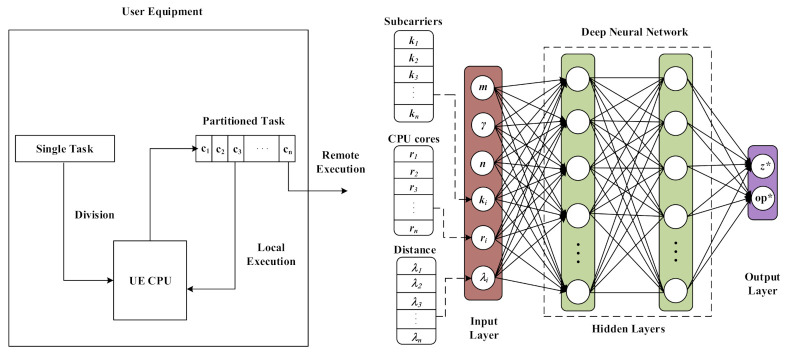
The proposed system model with the partitioning concept.

We consider a partial offloading technique [42], where a UE divides a single task into *n* components, $C=\{c_{1},c_{2},c_{3},...,c_{n}\}$. We assume that the number of components per task, *n*, is known before partitioning. Each component, $c_{i}\in C$, i=1,2,...,n, can be executed locally or offloaded to MES in a sequential manner, as shown in Figure 1. The components of a task can be modeled as a directed graph, as in [38]. To represent this mathematically, we introduce a binary variable $e_{i}\in \left[1,0\right]$. If $e_{i}=0$, $c_{i}$ executes locally on UE, otherwise $c_{i}$ executes remotely on MES. Therefore, we develop the models of both local and remote executions. The input data of $c_{i}$ is represented as $\mu_{i}$ and after the execution of $c_{i}$ the output resultant data is represented as $\rho_{i}$. The number of central processing unit (CPU) cycles required to process $c_{i}$ is $\alpha_{i}$, which depends on the value of $\mu_{i}$ as $\alpha_{i}=\eta \times \mu_{i}$, where η represents the number of CPU cycles per bit.

### 2.1. Local Execution Model ($e_{i}=0$)

We consider heterogeneous computing capability of UEs. Therefore, the total delay, $d_{li}$, for executing $c_{i}$ locally can be written as:

(1) $$d_{li}=\frac{\alpha_{i}}{f_{ui}},$$

where $f_{ui}$ is the CPU frequency that a UE selects to process $c_{i}$. Similarly, the energy consumption due to local execution of $c_{i}$, $E_{li}$, can be written as:

(2) $$E_{li}=d_{li}\epsilon f_{ui}^{\zeta },$$

where ϵ is a constant that depends on the average switch capacitance and average activity factor of UE. ζ is a constant of UE having a value greater than 2 [32].

### 2.2. Remote Execution Model ($e_{i}=1$)

An UE can upload a component $c_{i}$ to the MES for execution. If the network utilizes orthogonal frequency-division multiple access (OFDMA) then we can assume that the bandwidth, *B*, for transmission is divided into *K* subcarriers. The notations used in this paper are given in Table 2. For transmission and reception of $\mu_{i}$ and $\rho_{i}$, respectively, the available subcarriers are represented by $k_{i}\in \left\{1,2,3,...,K\right\}$, where *K* is the maximum available subcarriers. Similarly, $r_{i}\in \left\{0,1,2,...,R\right\}$, represents the number of CPU cores used in the processing of $c_{i}$ and *R* represents the maximum number of CPU cores at MES. $r_{i}=0$ implies that for component $c_{i}$ there is no CPU available and the system is busy. We consider additive white Gaussian noise for uplink and downlink data rates [37], which can be written as follows.

(3) $$v_{upi}=\frac{k_{i}}{K}B\log_{2}\left(1+\frac{p_{ui}^{t}{\left|h_{up}\right|}^{2}}{\Gamma \left(g_{up}\right)\lambda_{i}^{\theta }N_{o}}\right),$$

(4) $$v_{dli}=\frac{k_{i}}{K}B\log_{2}\left(1+\frac{p_{si}{\left|h_{dl}\right|}^{2}}{\Gamma \left(g_{dl}\right)\lambda_{i}^{\theta }N_{o}})\right),$$

where $v_{upi}$ and $v_{dli}$ are the uplink and downlink data rates for component $c_{i}$, respectively, while $p_{ui}^{t}$ and $p_{si}$ are the transmitting powers of UE and MES, respectively, for $c_{i}$. $h_{up}$ and $h_{dl}$ are the channel fading coefficients while $g_{up}$ and $g_{dl}$ represent the bit error rates, for uplink and downlink, respectively. $\lambda_{i}$ is the distance between UE and MES. θ is the path loss exponent and $N_{o}$ is the noise power. $\Gamma \left(g_{dl}\right)=\frac{-2\log(5g_{dl})}{3}$ gives the signal to noise ratio (SNR) margin to meet the required bit error rate. The delay due to transmission, execution, and reception for $c_{i}$ can be calculated, respectively, as follows.

(5) $$d_{ti}=\frac{\mu_{i}}{v_{upi}},$$

(6) $$d_{ei}=\frac{\alpha_{i}}{r_{i}f_{s}},$$

(7) $$d_{ri}=\frac{\rho_{i}}{v_{dli}},$$

where $d_{ti}$, $d_{ei}$, and $d_{ri}$, are the delays due to transmission of input data $\mu_{i}$, execution of $\alpha_{i}$, and reception of output data $\rho_{i}$, respectively. $f_{s}$ is the frequency of MES’s CPU. Similarly, the propagation delay, when $c_{i}$ is executed remotely, can be calculated as $d_{pi}=\frac{\lambda_{i}}{c}$, where *c* is the speed of light. Hence, for $c_{i}$ component, the total remote execution delay, $d_{oi}$, can be formulated as:

(8) $$d_{oi}=d_{ti}+d_{ei}+d_{ri}+2d_{pi}.$$

Here, $2d_{pi}$ is added because the propagation delay is considered for both sides of communication, transmission of $\mu_{i}$, and reception of $\rho_{i}$. The energy consumption due to remote execution of $c_{i}$, $E_{oi}$, can be calculated as $E_{oi}=E_{ti}+E_{ri}$, where $E_{ti}$ is the energy consumption due to transmission of the input data, $\mu_{i}$, to MES, and $E_{ri}$ is the energy consumption due to reception of the output data, $\rho_{i}$. These energy consumptions can be calculated as:

(9) $$E_{ti}=d_{ti}p_{ui}^{t},$$

(10) $$E_{ri}=d_{ri}p_{ui}^{r},$$

where $p_{ui}^{r}$ is the received power at UE when $\rho_{i}$ data is received. Using (3) and (5), we can derive $E_{ti}$ as:

(11) $$E_{ti}=\frac{\mu_{i}\;K}{k_{i}B\log_{2}\left(1+\frac{p_{ui}^{t}{\left|h_{up}\right|}^{2}}{N_{o}\Gamma \left(g_{up}\right)\lambda_{i}^{\theta }}\right)}p_{ui}^{t}.$$

Similarly, using (4) and (7), we can write:

(12) $$E_{ri}=\frac{\rho_{i}\;K}{k_{i}B\log_{2}\left(1+\frac{p_{si}{\left|h_{dl}\right|}^{2}}{N_{o}\Gamma \left(g_{dl}\right)\lambda_{i}^{\theta }}\right)}p_{ui}^{r}.$$

### 2.3. Cost Function

The conventional optimization techniques and cost function with constraints have high computational overhead and algorithm complexity [43]. Therefore, we need to formulate a comprehensive cost function, considering all important parameters, for generating a training dataset only. The algorithm complexity for generating a training dataset through such a comprehensive cost function is also high however the computation during training phase only occurs once. After the training phase the trained DNN has constant complexity O(1) [41]. Our comprehensive cost function depends on delays and energy consumptions due to execution, transmission, reception, and task-division for partial offloading. Furthermore, the proposed cost function also considers the propagation delay, radio resources, and computing resources. In the partitioning process, if the number of components increases, the time delay and energy consumption due to task-division-process also increase. Therefore, the time delay, $d_{di}$, due to task-division per component can be written as:

(13) $$d_{di}≜f_{1}\left(n\right)=\frac{(n-1)\tau }{n},$$

where τ is the time in which the UE can divide a task in two components. Similarly, the energy consumption, $E_{di}$, per component due to partitioning of the task can be written as:

(14) $$E_{di}=d_{di}\epsilon f_{ui}^{3}.$$

We can write the equation for the total delay, $d_{i}$, for $c_{i}$ as:

(15) $$d_{i}=\left\{\begin{array}{cc} d_{li}+d_{di}, & e_{i}=0,\\ d_{oi}+d_{di}, & e_{i}=1. \end{array}\right.$$

Similarly, the total energy consumption, $E_{i}$, due to $c_{i}$ can be written as:

(16) $$E_{i}=\left\{\begin{array}{cc} E_{li}+E_{di}, & e_{i}=0,\\ E_{oi}+E_{di}, & e_{i}=1. \end{array}\right.$$

The cost function $f_{c}\left(c_{i},e_{i}\right)$ can be written as:

(17) $$f_{c}\left(c_{i},e_{i}\right)=\left\{\begin{array}{cc} f_{cl}\left(c_{i}\right), & e_{i}=0,\\ f_{co}\left(c_{i}\right), & e_{i}=1. \end{array}\right.$$

In (17), $f_{cl}\left(c_{i}\right)$ represents the local cost when $c_{i}$ executes locally on UE and $f_{co}\left(c_{i}\right)$ is the remote cost when $c_{i}$ executes remotely on MES. $f_{cl}\left(c_{i}\right)$ can be calculated as:

(18) $$f_{cl}\left(c_{i}\right)=\delta_{1}\left(\frac{d_{li}+d_{di}}{d_{max}}\right)+\delta_{2}\left(\frac{E_{li}+E_{di}}{E_{max}}\right),$$

where $\delta_{1}$ and $\delta_{2}$ are the weighting coefficients by which we can change the contribution and priority of delay and energy consumption in the cost function, respectively. $d_{max}$ is the deadline time for a whole task to execute, and it can be calculated as the average value of delays for different tasks (the delay of a single task is the sum of delays for all *n* components per task). $E_{max}$ is the maximum energy of UE’s battery. Similarly, $f_{co}\left(c_{i}\right)$ can be written as:

(19) $$\begin{array}{cc} f_{co}\left(c_{i}\right)= & \;\delta_{3}\left[d_{ti}\left(1-e_{i-1}\right)+d_{ei}+d_{ri}+d_{pi}\left(2-e_{i-1}\right)+d_{di}\right]\\ \; & +\delta_{4}\left[E_{ti}\left(1-e_{i-1}\right)+E_{ri}+E_{di}\right]+\delta_{5}f_{2}\left(r_{i}\right)+\delta_{6}f_{3}\left(k_{i}\right), \end{array}$$

where $\delta_{3}$, $\delta_{4}$, $\delta_{5}$, and $\delta_{6}$ are the weighting coefficients. The values of these coefficients can be calculated according to the priority of time delay, energy consumption, radio resources, and computational resources in the cost function. For example, if the importance of time delay is higher than energy consumption then $\delta_{3}$ should be greater than $\delta_{4},\;\delta_{5},$ and $\delta_{6}$. However, the sum of all coefficients must be equal to 1. We multiply $\left(1-e_{i-1}\right)$ with $d_{ti}$ and $E_{ti}$, because if the previous component $c_{i-1}$ is executed remotely then $e_{i-1}=1$. It means the data for $c_{i}$ is available at MES and we do not need to transmit it again. Therefore, the delay and energy consumption due to transmission must be zero. Similarly, $d_{pi}\left(2-e_{i-1}\right)$ gives propagation delay only for reception if $e_{i-1}=1$. $f_{2}\left(r_{i}\right)$ and $f_{3}\left(k_{i}\right)$ give the cost due to used CPU cores and subcariers, respectively, and can be given as $f_{2}\left(r_{i}\right)=r_{i}/R$ and $f_{3}\left(k_{i}\right)=k_{i}/K$.

## 3. The Proposed Deep Learning Approach

In the proposed work, first we divide a task into *n* components and then using the partial offloading technique, the UE offloads some of the components to MES and some of the components are executed on UE. However, for a task of size *m* there are ${}^{m}\;Z_{n}$ possible partitioning options and $2^{n}$ possible offloading options, therefore, to find the option with minimum cost, the complexity of the algorithm becomes $O({}^{m}\;Z_{n}2^{n})$. To avoid this computation overhead and high complexity, we generate a training dataset using our comprehensive mathematical model to find the cost for all ${}^{m}\;Z_{n}2^{n}$ options. In Algorithm 1, we consider all possible partitions (partition matrix, PR) and all possible partial offloading policies (offloading policies matrix, OP) for a task of size *m* with a constant γ. We select the option with minimum cost and store its corresponding input data, partitioning option, and offloading policy as a training dataset for different task sizes. To consider the dynamics of the system, we take the input parameters (frequency of UE, tranmiting power of UE, distance between UE and MES, subcarriers, computing resources of MES, number of components, division resolution, and task size) as uniform random distribution for different datasets. This training dataset is used to train the DNN and reduce the complexity to O(1) of the trained DNN. The trained DNN has the ability to address the dynamics of the system because in the training dataset we consider all the dynamics in the input data parameters. Our proposed technique considers the comprehensive cost function to minimize the energy consumption and service delay, therefore, we name the proposed technique as comprehensive and energy efficient deep-learning-based offloading technique (CEDOT). There are three types of layers in the DNN [44,45], namely input layer, hidden layer, and output layer. The input layer consists of the information about the size of task, division resolution (γ), number of components, distance from MES, available computing resources of MES, and network status, as shown in Algorithm 1. The two hidden layers consist of 100 neurons each. The output layer gives the information about the best option for division $\left(z^{*}\right)$ and offloading decision for each component represented by $op^{*}$. The rectified linear unit (ReLU) and Softmax activation functions [46,47] are used for two hidden layers and an output layer, respectively. The trained DNN is tested on unseen data to calculate the accuracy of the proposed technique. We achieve more than 70% accuracy for different sets of test data.

The following benchmark techniques are considered for comparison with CEDOT: (i) Total offloading technique (TOT) [37], (ii) random offloading technique (ROT) [37], (iii) deep learning-based offloading technique (DOT) [37], and (iv) energy efficient deep learning-based offloading technique (EEDOT) [38]. TOT offloads all components to MES without considering other partial offloading options. ROT selects any offloading decision at random. DOT considers all available $2^{n}$ offloading decisions and train a DNN with the training dataset containing the minimum cost but the energy consumption and component size are ignored in the cost function. EEDOT considers the partial offloading technique with random size of components and does not consider the partitioning in the cost function. None of the above techniques consider the delay and energy consumption of task-division-process in their approaches. Similarly, none of them consider the energy consumption of UE in the cost function.

The proposed technique (CEDOT) finds the partitioning and offloading decisions with minimum cost, in terms of service delay and energy consumption, simultaneously, using the trained DNN which is trained on a comprehensive dataset generated from the comprehensive mathematical model.
**Algorithm 1** Partial Offloading with Partitioning**Input:** {$m,\;\gamma ,\;n,\;r_{i},\;k_{i},\;\lambda_{i}$}**Output:** {$z^{*},\;op^{*}$}1:PR← Matrix of possible partitions2:OP← Matrix of possible offloading policies3:**for**$k=1:{}^{M}\;Z_{i}$**do**4:    P(k)←PR(k,:)5:    **for** $l=1:2^{i}$ **do**6:        e(l)←OP(l,:)7:        **for** j=1:i **do**8:           **if** (e(j)=0) **then**9:               $cost\left(j\right)\leftarrow f_{lc}$ using (18)10:           **else**11:               $cost\left(j\right)\leftarrow f_{oc}$ using (19)12:           **end if**13:        **end for**14:        $cost_{1}\left(l\right)\leftarrow$sum(cost)15:    **end for**16:    $\left[index,cost_{2}\left(k\right)\right]\leftarrow min\left(cost_{1}\right)$17:    ${\mathrm{OP}}_{1}\left(k\right)\leftarrow \mathrm{OP}\left(index,:\right)$18:**end for**19:$\left[index,cost_{minimum}\right]\leftarrow min\left(cost_{2}\right)$20:$op^{*}\leftarrow {\mathrm{OP}}_{1}\left(index,:\right)$21:$z^{*}\leftarrow \mathrm{PR}\left(index,:\right)$22:Save input data: $input\leftarrow \{m,\;\gamma ,\;n,\;r_{i},\;k_{i},\;\lambda_{i}\}$23:Save output data: $labels\leftarrow \{z^{*},op^{*}\}$24:Train the DNN: Trained_DNN←train(input,labels)

## 4. Simulation Results and Discussion

The simulation environment used is MATLAB (R2019a) running on an Intel Core i7 processor with a clock rate of 3.4 GHz. In the simulation, all techniques, except TOT, divide a task of size *m* in six components, which are executed in a sequence on UE or through MES. All random variables are independent for different components. The number of raw data that is used to generate the training dataset is 40,000, which means that we execute 40,000 tasks of different sizes, uniformly distributed in [0.1 1.5] GB, independently through our proposed algorithm and save the output with minimum cost as labels for corresponding input data. The $f_{ui}$, $p_{ui}^{t}$, $\lambda_{i}$, $k_{i}$, and $r_{i}$ are taken as uniformly distributed in [0.1, 1] GHz, [0.8 1.25] Watt, [3 800] m, [1 256], and [0 16], respectively. The number of CPU cycles to process one bit of data is taken as 737.5 cycles/bit. The effective switching capacitance factor is taken as $10^{-27}$. The simulation parameters are given in Table 3.

After getting the training dataset, we also generate a test dataset of size 10,000 to check the accuracy of trained DNN for unseen data. There are 21 neurons in the input layer; one for task size, one for division resolution, one for number of components per tasks, six for random distances during mobility of UEs in execution of each component, six for subcarriers during the execution of each component, and six for CPU cores assigned to each components. Similarly, six neurons for offloading policy and six neurons for partitioning are reserved in the output layer. The ReLU and Softmax activation function are used for the two hidden layers and the output layer, respectively.

**Figure 2:**
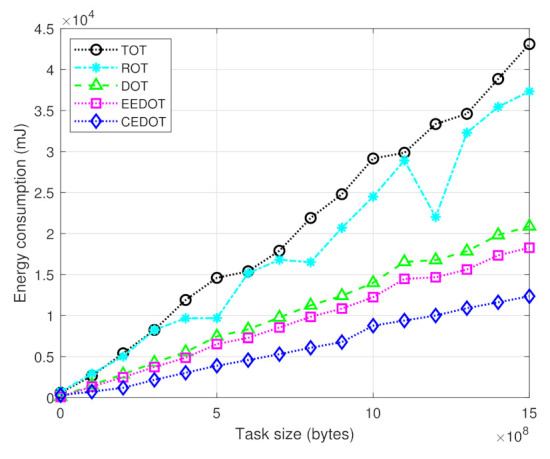
Energy consumption for different task sizes.

Figure 2 presents the comparison of energy consumption of a UE with a varying task size. The energy consumption of CEDOT is minimum because it considers the suitable component size along with the offloading policy having minimum cost. For different component sizes, the energy consumption varies with different offloading policies. However, the proposed technique selects the offloading policy with minimum cost of portioning and offloading.

**Figure 3:**
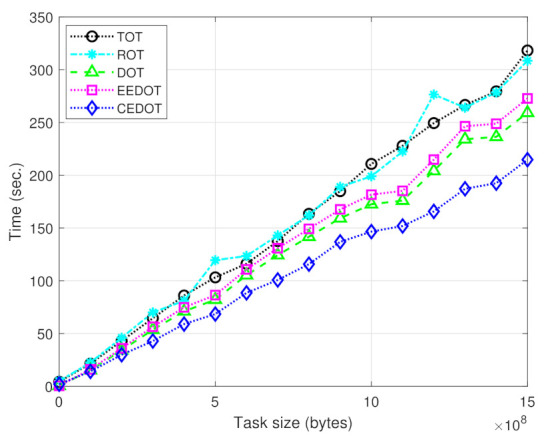
Service delay for different task sizes.

Figure 3 shows the service delay versus task size. Here, we can observe better performance of CEDOT because of the proposed comprehensive cost function. The benchmark techniques do not consider the delay and energy consumption due to task-division for partial offloading in the cost function, which is not a realistic approach. However, the proposed technique considers various types of realistic delays in the cost function and finds minimum values for various types of delays. The benchmark techniques select components with random sizes and also ignore them in the cost function.

**Figure 4:**
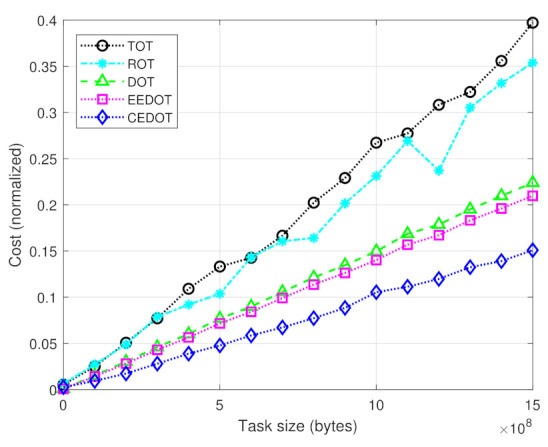
Cost for different task sizes.

Figure 4 shows the normalized cost of all the techniques with a varying size of tasks. The cost of the proposed technique is minimum because it selects the minimum value among ${}^{m}\;Z_{n}2^{n}$ possible values of the cost. However, the other benchmark techniques ignore this fact and select the cost value depending only on time or energy consumption.

**Figure 5:**
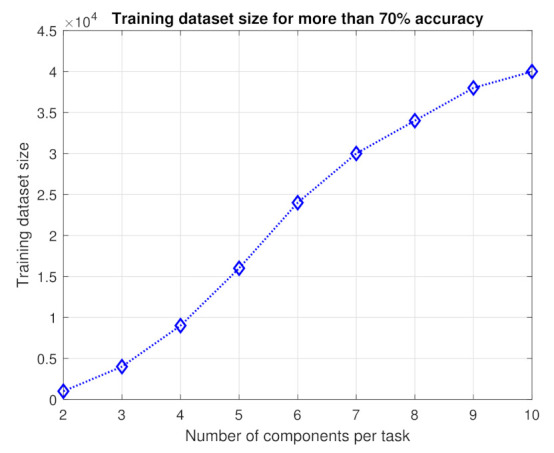
Training dataset size for different number of components with more than 70% accuracy.

Figure 5 presents the size of training dataset along with a different number of components per task. As the number of components per task increases, the complexity of the decision boundaries increases. Therefore, to achieve more than 70% accuracy, we need to change the size of the training data set, as depicted in Figure 5.

**Figure 6:**
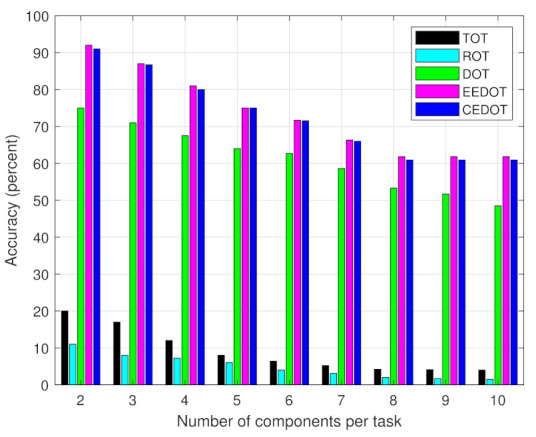
DNN accuracey for different number of components per task.

Similarly, the effect of the number of components per task on accuracy is shown in Figure 6. As the number of components per task increases, the possible number of offloading policies (which is $2^{n}$) increases. Therefore, the chances of selecting the offloading policy with minimum cost decreases and the performance of TOT and ROT decreases. While for the other techniques using deep learning-based approaches, the complexity between constant input and output data increases, the accuracy of CEDOT, EEDOT, and DOT decreases as the number of components per task increases. However, the performance of the proposed work is higher than all the other techniques and is almost equal to EEDOT with the advantage of low energy consumption and time delay for computational offloading and partitioning.

**Figure 7:**
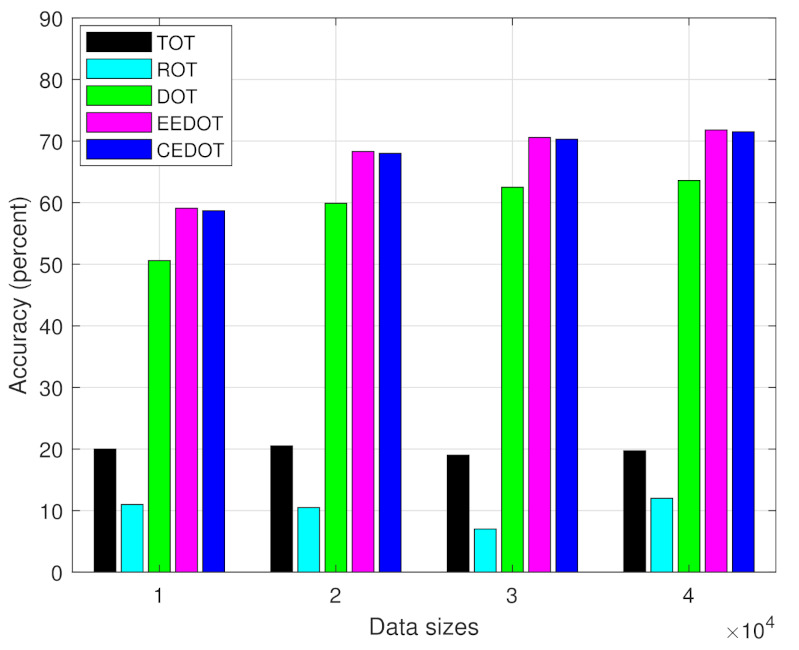
Comparison of the DNN accuracy for different sizes of training dataset.

Figure 7 shows the accuracy of the trained DNN along with varying size of training dataset. We can observe that the accuracy increases as the size of training dataset increases because large datasets means more data which provides more information to the DNN. The performance of CEDOT and EEDOT is better and almost equal, however, the proposed technique solves the computational offloading and partitioning jointly while EEDOT ignores the partitioning of the task.

**Figure 8:**
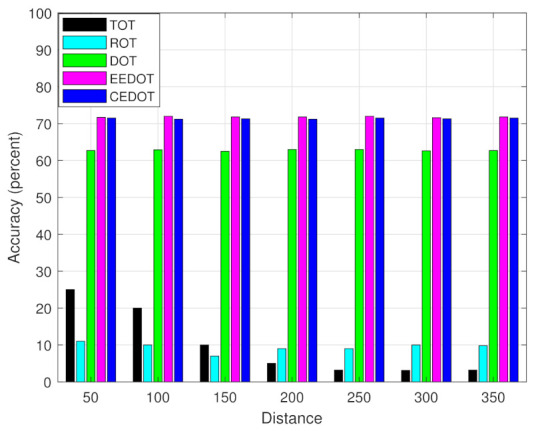
Comparison of the DNN accuracey with respect to distance.

Figure 8 shows the accuracies of different techniques along with varying distance between MES and UE. CEDOT, EEDOT, and DOT have almost constant accuracy for different distances which means the accuracy of the DNN is not affected by the mobility of UEs. TOT has a decreasing behavior as the distance increases between MES and UE. It is because at lower distances the chances of offloading all components to MES are high since the transmission and reception cost is minimum. However, the accuracies of TOT and ROT are lower as compared to the other techniques because there is no learning mechanism used in TOT and ROT.

### Discussion

All the simulation results show a better performance of CEDOT because of its comprehensive mathematical model for cost function. In the cost function, we consider all the important and realistic parameters to calculate time delay and energy consumption for the completion of the task. Most of the related works have not considered the task-division-cost in the mathematical models and assumed random values for each component of a task. However, there must be some energy consumption and time delay due to a task division process which we have considered in our proposed work. For the sake of better comparison we also include the cost of division process in all the benchmark techniques. The comprehensive mathematical model for cost function gives the more authentic training dataset for DNN. Therefore, the other techniques select the offloading policy with higher energy consumption and time delay as compared to the CEDOT because, their cost function and mathematical model are not that realistic and comprehensive. By considering more and realistic parameters in the cost function, it increases the accuracy of DNN because the relation and linkages between input data and labels become stronger and clearer. The proposed technique selects the offloading policy and partitioning with minimum cost which means that the optimal offloading policy selected by CEDOT will consume minimum energy with faster execution of a task.

We can observe that the accuracy of the deep learning approaches increases by using a larger dataset, while the accuracy of the TOT and ROT is not affected by the size of data since there is no learning mechanism involved. The offloading decisions of TOT are constant and ROT has a random nature of selection without any mechanism. The accuracy of all the techniques decreases as the number of components per task increases. The decline in accuracy of TOT and ROT, with a number of components per task, is due to the increase in the number of offloading policies and therefore, the probability of selecting policy with minimum cost is decreased. The decline in the accuracy of deep learning-based approaches is because of the decision boundaries becoming more complex with an increase in the number of components per task. The accuracy of only TOT is affected by distance, the rest of the techniques have constant accuracy during the mobility of UEs. Since, at lower distances the cost of offloading all components become smaller, therefore, TOT has higher accuracy at lower distances.

A limitation of our proposed model is that it cannot find the optimal number of components per task. It is necessary to know the number of components per task before the execution of the task. Similarly, the proposed technique works only for the applications having sequential execution of components due to dependencies of components. For example, if the application has callbacks or loops through previous components, then the proposed technique cannot handle that scenario.

## 5. Conclusions

In this paper, we proposed a comprehensive cost function for energy efficient computational offloading in MEC. A supervised deep learning approach was used to find the partitioning of a task in *n* components along with an offloading policy having minimum cost in terms of energy consumption and time delay. Our cost function takes into account various energy consumptions and delays due to task-division, transmission, execution, and reception. The proposed approach comprehensively models the real environment which is better suited for implementation in practical scenarios. The end-user can use the trained DNN to find the offloading policy and partitioning for *n* number of components per task with minimum cost. Since, the cost function depends on both energy consumption and time delay, therefore, the end-user will consume minimum energy with a faster decision process in selecting the best offloading policy for *n* number of components per task. The simulation results demonstrate improved energy consumption and service delay with more than 70% accuracy of the DNN. For future work, DNN can be trained to simultaneously optimize the number of components per task, the size of each component, and offloading policy for all types of applications.

## Figures and Tables

**Table 1:** Summary of the related work.

Techniques	Considers Service Delays?					Considers Energy Consumption?			Task Partitioning Considered?	Multi-User Multi-Server Considered?	Deep Learning Approach?
	Transmission $d_{\mathrm{ti}}$	Execution $d_{\mathrm{ei}}$, $d_{\mathrm{li}}$	Reception $d_{\mathrm{ri}}$	Partitioning $d_{\mathrm{di}}$	Propagation $d_{\mathrm{pi}}$	Transmission $E_{\mathrm{ti}}$, $E_{\mathrm{li}}$	Reception $E_{\mathrm{ri}}$	Partitioning $E_{\mathrm{di}}$			
MDP-based VIA Technique [16]	Yes	Yes	Yes	No	No	No	No	No	No	Yes	No
Reliability-aware Offloading [17]	Yes	Yes	Yes	No	No	Yes	Yes	No	No	Yes	No
Traditional Optimization Techniques [18,19]	Yes	Yes	No	No	No	Yes	No	No	No	No	No
Energy Harvesting Techniques [20,21,22,25,26,27]	Yes	Yes	No	No	No	Yes	No	No	No	No	No
Genetic Algorithm -based Offloading [30]	Yes	Yes	No	No	No	Yes	No	No	No	Yes	No
Offloading of DNN-driven Applications [31]	Yes	Yes	No	No	Yes	Yes	No	No	No	Yes	No
Offloading for OCR Case [32]	Yes	Yes	No	No	Yes	No	No	No	Yes	No	No
Game Theoretic Approach [34]	No	No	No	No	No	Yes	Yes	No	No	Yes	No
Energy Efficiency-based Offloading [35,37,41]	Yes	Yes	No	No	No	No	No	No	No	Yes	Yes
Cost Function-based Offloading [36,38]	Yes	Yes	Yes	No	No	Yes	Yes	No	No	Yes	Yes
Cost Function-based Offloading [39,40]	Yes	Yes	No	No	No	Yes	No	No	No	No	Yes
Our Proposed Technique (CEDOT)	Yes	Yes	Yes	Yes	Yes	Yes	Yes	Yes	Yes	Yes	Yes

**Table 2:** List of notations.

Notations	Meaning
$\alpha_{i}$	Number of CPU cycles to process $c_{i}$
*B*	Transmission bandwidth
*C*	Set off components per tasks
$c_{i}$	*i*th component
$d_{li}$	Total required delay to execute $c_{i}$ locally
$d_{ti}$	Required delay for transmission of $c_{i}$
$d_{ei}$	Required delay for execution of $c_{i}$
$d_{ri}$	Required delay for reception of $c_{i}$
$d_{pi}$	Propagation delay for $c_{i}$
$d_{oi}$	Total remote execution delay for $c_{i}$
$d_{di}$	Delay due to division process per component
$E_{di}$	Energy consumption due to division process per component
$E_{li}$	Total energy consumption to execute $c_{i}$ locally
$E_{oi}$	Total remote energy consumption for $c_{i}$
$E_{ti}$	Transmission energy consumption for $c_{i}$
$E_{ri}$	Reception energy consumption for $c_{i}$
ϵ	Average switch capacitance and activity factor
$e_{i}$	Binary offloading decision variable
η	Number of CPU cycles per bit
$f_{cl}\left(c_{i}\right)$	Local cost for component $c_{i}$
$f_{co}\left(c_{i}\right)$	Remote cost for component $c_{i}$
$f_{s}$	CPU frequency of MES
$f_{ui}$	CPU frequency of UE
$\delta_{1}$, $\delta_{2}$	Weighting coefficients for local cost function
$\delta_{3}$, $\delta_{4}$	Weighting coefficients for remote cost function
γ	Division resolution in partitioning
$h_{dl}$, $h_{up}$	Channel fading coefficients for downlink, uplink
*K*	Maximum available subcarriers
$k_{i}$	Number of subcarriers assigned to $c_{i}$
$\lambda_{i}$	Distance between UE and MES
*m*	Task size
$\mu_{i}$	Input data size of $c_{i}$
$N_{o}$	Noise power
*n*	Number of components per tasks
OP	Matrix of possible offloading policies
$op^{*}$	Optimal partitioning
PR	Matrix of possible partitions
$p_{si}$	Transmitting power of MES
$p_{ui}^{t}$	Transmitting power of UE
$p_{ui}^{r}$	Receiving power of UE
*R*	Maximum CPU cores of MES
θ	Path loss exponent
τ	Required delay to divide a task into two components
$r_{i}$	Number of CPU cores of MES assigned to $c_{i}$
$\rho_{i}$	Output data size of $c_{i}$
$v_{dli}$	Downlink data rate
$v_{upi}$	Uplink data rate
$z^{*}$	Optimal offloading policy

**Table 3:** Simulation parameters.

Parameter	Value	Parameter	Value
*B*	0.5 MHz	*R*	256
$E_{max}$	800 J	$p_{ui}^{t}$	1.2 W
*K*	16	$p_{ui}^{r}$	0.8 W
$d_{max}$	300 s	$\delta_{1}$	0.6
γ	200 MB	$\delta_{2}$	0.4
ϵ	$10^{-27}$	$\delta_{3}$	0.5
η	737.5 cycles/bit	$\delta_{4}$	0.3
$N_{o}$	−174 dBm/Hz	$\delta_{5}$	0.1
θ	3	$\delta_{6}$	0.1
